# Supplementary material for: CLIQ-BID: A method to quantify bacteria-induced damage to eukaryotic cells by automated live-imaging of bright nuclei
Source: Sci Rep. 2018 Jan 8;8:5. doi: 10.1038/s41598-017-18501-9 (PMC5758606; doi:10.1038/s41598-017-18501-9)
Supplement: Supplementary file 1 — Supplementary information [file 41598_2017_18501_MOESM1_ESM.pdf]

CLIQ-BID: A method to quantify bacteria-induced damage to eukaryotic cells by automated live-imaging of bright nuclei

Yann Wallez, Stéphanie Bouillot, Emmanuelle Soleilhac, Philippe Huber, Ina Attrée, Eric Faudry

Supplementary information

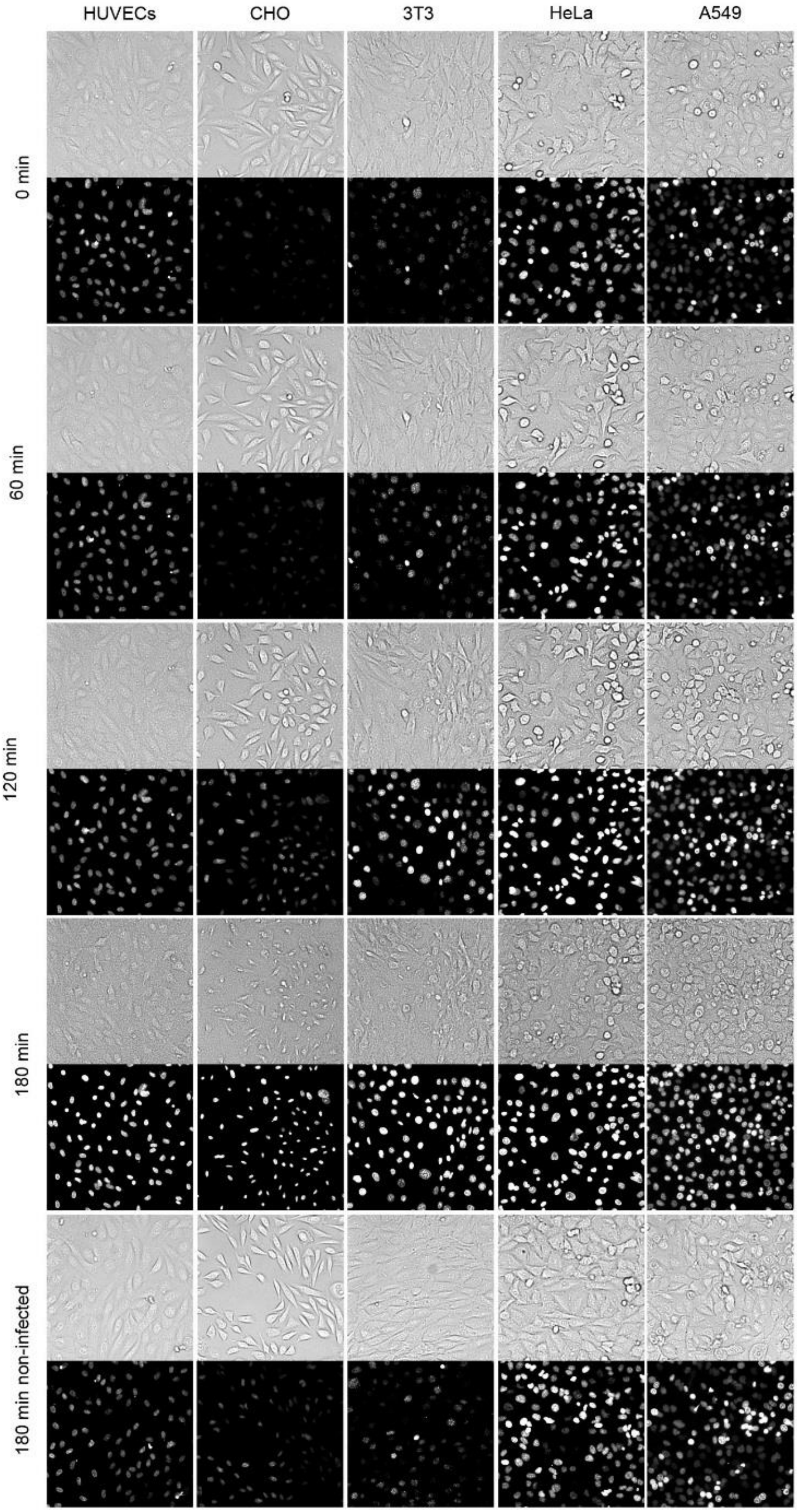

Supplementary Figure S1: *Pseudomonas aeruginosa*-induced cell damages result in smaller and brighter cell nuclei in five different cell types. Human primary endothelial cells (HUVECs), CHO, 3T3, HeLa and A549 cells were infected with *P. aeruginosa* and monitored at different stages of infection by live-imaging microscopy with vital-Hoechst nuclear stain. Cell surface (transmitted light) and cell nuclei (fluorescent labelling), upper and lower images respectively, in the same fields at one hour intervals. The last image set corresponds to cells that where not incubated with bacteria, after three hours.

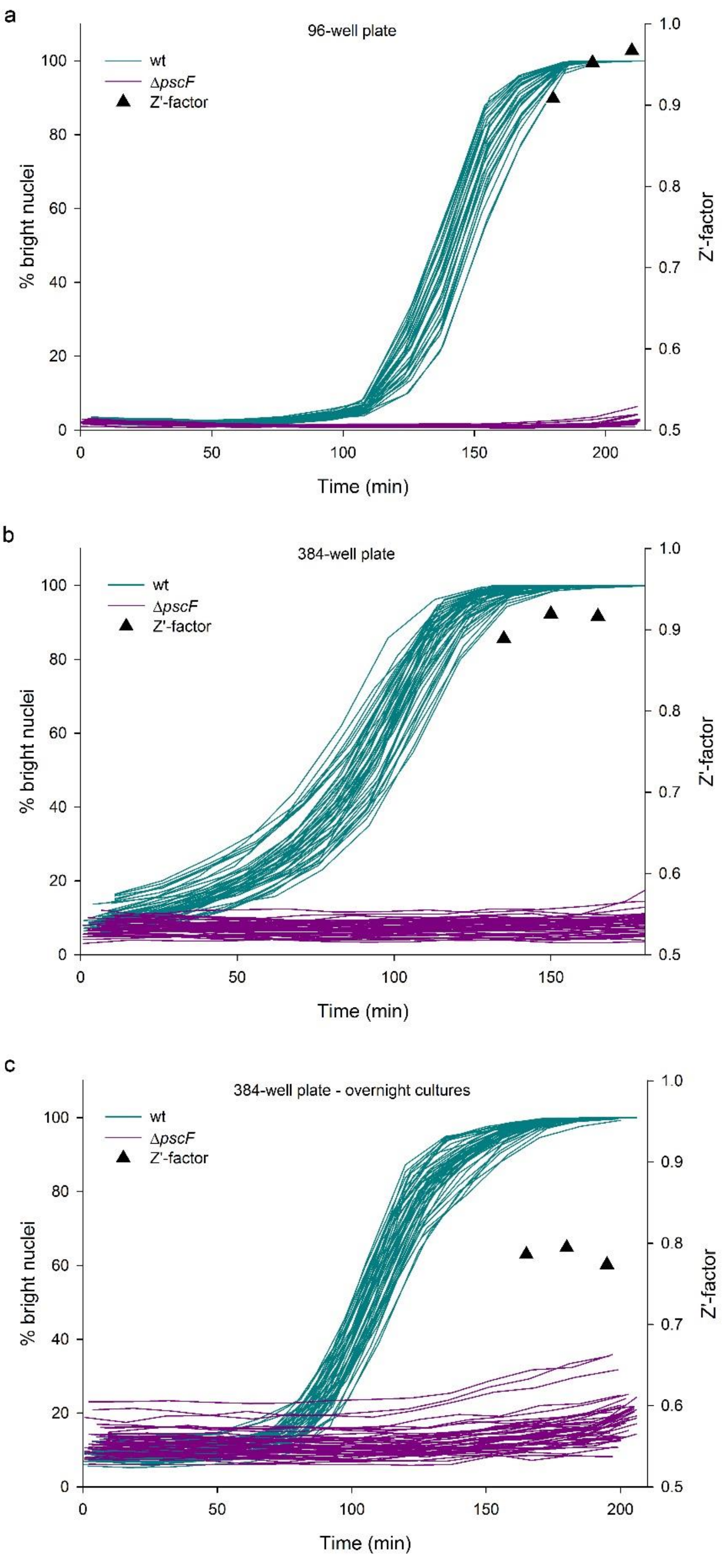

Supplementary Figure S2: Detection of bright nuclei is well-suited for HCS strategies. HUVECs were infected with *P. aeruginosa* wild-type strain or a strain deficient for the production of the T3SS needle subunit (Δ*pscF*), and the presence of bright nuclei was monitored by live cell imaging. Kinetics plots were superposed for the 48 replicates of each condition and the Z'-factor values were calculated for the last three time points. Cell infections were performed in 96- well plates (a) and 384-well plates (b and c).

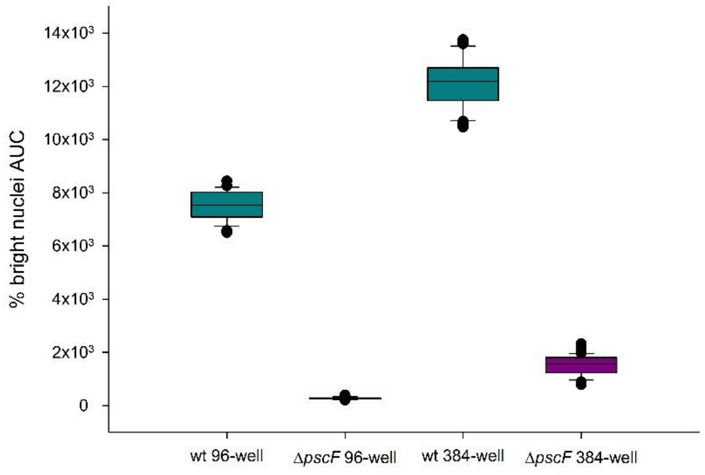

Supplementary Figure S3: HUVECs were infected with *P. aeruginosa* wild-type strain or a strain deficient for the production of the T3SS needle subunit (Δ*pscF*), and the presence of bright nuclei was monitored by live cell imaging in 96- well plates and 384-well plates. The Area Under the Curves (AUC) of the kinetics plots were subsequently derived and represented in box plots. Whiskers indicate the 10th and 90th percentiles; the top and bottom lines represent the 25th and 75th percentiles; the middle line and dots respectively show median and outliers.

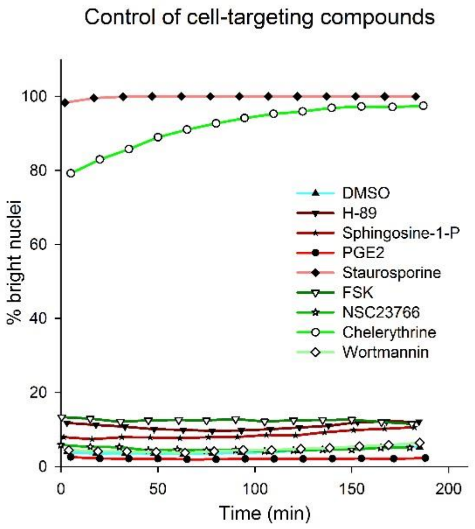

Supplementary Figure S4: Effect of cell-targeting compounds in the absence of bacteria**.** HUVECs were incubated with molecules targeting the eukaryotic cells or DMSO as a control. The kinetics of bright nuclei apparition was recorded by live-imaging and processed by the CLIQ-BID method.

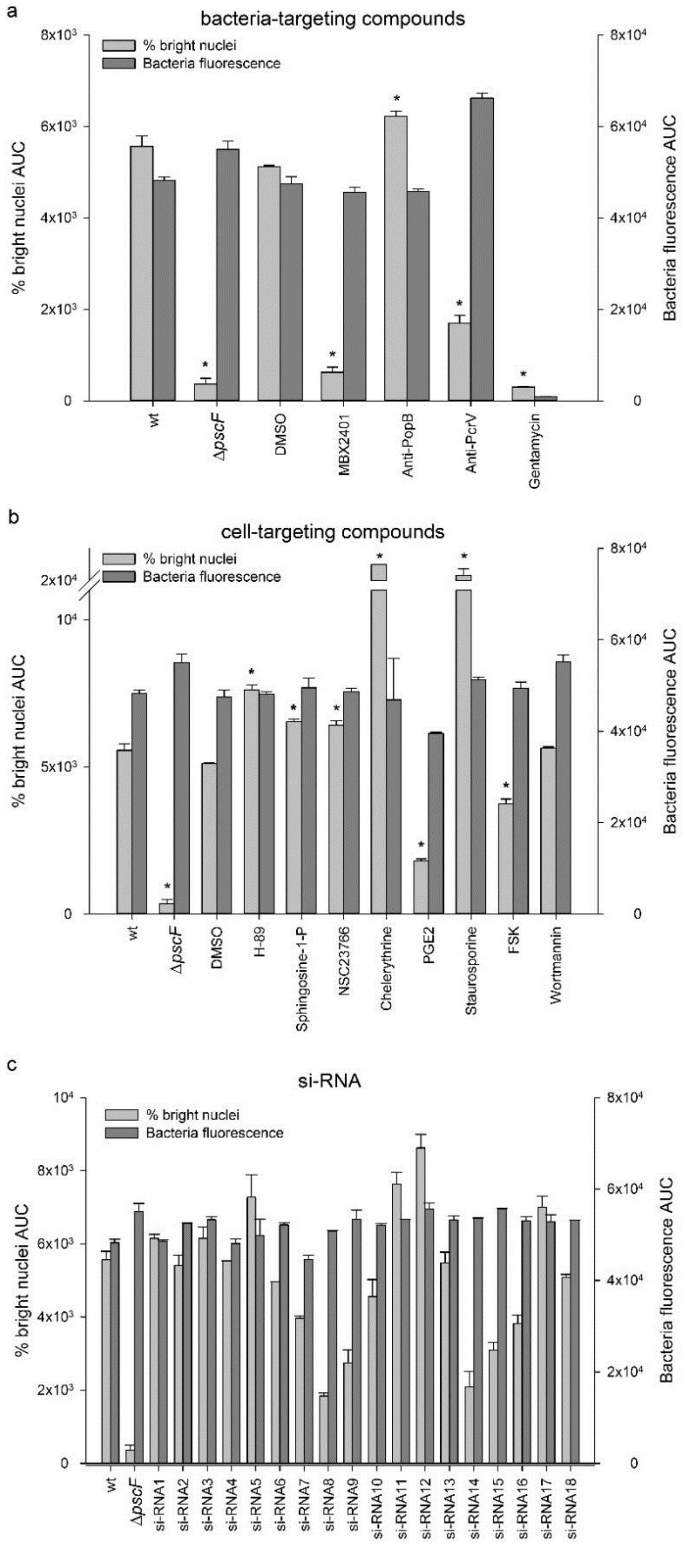

Supplementary Figure S5: Area Under the Curve analysis of compounds screening**.** HUVECs were infected with *P. aeruginosa* wild-type strain in the presence of molecules targeting the bacteria (a) or targeting the eukaryotic cells (b) and their respective controls. To assess the effect of siRNA transfection in the cells, HUVECs were transfected two days before infection (c). A strain deficient for the production of the T3SS needle subunit (Δ*pscF*) was used as control. The kinetics of bright nuclei apparition and bacteria growth were simultaneously recorded by live-imaging and the corresponding Area Under the Curves (AUC) were subsequently calculated. Error bars represent the standard deviation (n=3). Stars indicate statistically significant differences with the wild-type (wt) infection in the absence of molecules (one-way ANOVA, $P < 0.05$). For the sake of clarity, stars are not shown in the AUC plot corresponding to siRNAs.

	HUVEC 20x n=4					
Parameter	Mean nuclei area		Mean nuclei intensity		% bright nuclei*	
Time	0 min	180 min	0 min	180 min	0 min	180 min
Moy	375.88	272.73	1505.13	2897.60	4.22	90.08
Median	364.42	271.95	1439.61	2812.91	2.95	87.72
SD	23.82	3.43	158.53	188.57	3.60	6.58
CV (%)	6.34	1.26	10.53	6.51	85.47	7.30
Z' 180/0min	0.21		0.25		**0.64**	

	HUVEC 5x n=4					
Parameter	Mean nuclei area		Mean nuclei intensity		% bright nuclei*	
Time	0 min	180 min	0 min	180 min	0 min	180 min
Moy	31.47	21.44	1319.27	2107.52	8.81	90.96
Median	31.01	21.29	1320.08	2106.46	8.74	91.78
SD	1.46	1.06	33.49	55.33	0.58	6.30
CV (%)	4.63	4.92	2.54	2.63	6.57	6.93
Z' 180/0min	0.25		**0.66**		**0.75**	

*= % nuclei with intensity above a fixed threshold

Supplementary Table S1: Intensity thresholding and low magnification images better discriminate between infected and uninfected cells. Statistical comparison of quadruplicate wells analysis, considering the mean nuclei area, mean nuclei intensities and the percentage of nuclei with intensities above a fixed threshold obtained from the same image sets. HUVECs were infected for 3 hours with *P. aeruginosa* and images were obtained at 20x and 5x magnifications.

Statistical comparison of two quantification methods (n=30)

	% surface cleared by cells		% bright nuclei	
	wt	Δ*pscF*	wt	Δ*pscF*
**Mean**	35.53	13.82	97.04	1.67
**Median**	35.29	13.66	97.69	1.22
**SD**	2.91	3.33	2.09	1.35
**CV (%)**	8.18	24.08	2.15	81.10
**Z'**	0.14		0.89	

Supplementary Table S2: Statistical comparison of the "cell area" and "bright nuclei" quantification methods. Statistical comparison of 30 replicates, considering the area cleared by the cells after infection and the percentage of nuclei with intensities above a fixed threshold obtained from the same image sets. HUVECs were infected for 3 hours with *P. aeruginosa* wild-type strain or a strain deficient for the production of the essential T3SS needle subunit protein (Δ*pscF*).

P96 Area Under the Curve

	wt	D*pscF*
**Mean**	7,551	281
**Median**	7,543	277
**SD**	518	40
**CV (%)**	6.86	14.09
**Z'**	0.77	

P384 Area Under the Curve

	wt	D*pscF*
**Mean**	12,103	1,528
**Median**	12,190	1,568
**SD**	920	384
**CV (%)**	7.60	25.14
**Z'**	0.63	

Supplementary Table S3: Statistical comparison of the Area Under the Curves kinetics descriptor. Statistical comparison of 48 replicates wells analysis, considering the Area Under the Curves of the kinetics plots. HUVECs were infected with *P. aeruginosa* wild-type strain or a strain deficient for the production of the T3SS needle subunit (Δ*pscF*).

Supplementary Video S1: Human primary endothelial cells (HUVECs), CHO, 3T3, HeLa and A549 cells were infected with *P. aeruginosa* and monitored at different stages of infection by live-imaging microscopy with vital-Hoechst nuclear stain. Cell surface and cell nuclei were observed at 15 min intervals by transmitted light (upper images) and fluorescent labelling (lower images), respectively.
